# Behavior and Properties of Mature Lytic Granules at the Immunological Synapse of Human Cytotoxic T Lymphocytes

**DOI:** 10.1371/journal.pone.0135994

**Published:** 2015-08-21

**Authors:** Min Ming, Claudia Schirra, Ute Becherer, David R. Stevens, Jens Rettig

**Affiliations:** Center for Integrative Physiology and Molecular Medicine (CIPMM), Medical Faculty, Saarland University, 66421, Homburg, Germany; J. Heyrovsky Institute of Physical Chemistry, CZECH REPUBLIC

## Abstract

Killing of virally infected cells or tumor cells by cytotoxic T lymphocytes requires targeting of lytic granules to the junction between the CTL and its target. We used whole-cell patch clamp to measure the cell capacitance at fixed intracellular [Ca^2+^] to study fusion of lytic granules in human CTLs. Expression of a fluorescently labeled human granzyme B construct allowed identification of lytic granule fusion using total internal reflection fluorescence microscopy. In this way capacitance steps due to lytic granule fusion were identified. Our goal was to determine the size of fusing lytic granules and to describe their behavior at the plasma membrane. On average, 5.02 ± 3.09 (mean ± s.d.) lytic granules were released per CTL. The amplitude of lytic granule fusion events was ~ 3.3 fF consistent with a diameter of about 325 nm. Fusion latency was biphasic with time constants of 15.9 and 106 seconds. The dwell time of fusing lytic granules was exponentially distributed with a mean dwell time of 28.5 seconds. Fusion ended in spite of the continued presence of granules at the immune synapse. The mobility of fusing granules at the membrane was indistinguishable from that of lytic granules which failed to fuse. While dwelling at the plasma membrane lytic granules exhibit mobility consistent with docking interspersed with short periods of greater mobility. The failure of lytic granules to fuse when visible in TIRF at the membrane may indicate that a membrane-confined reaction is rate limiting.

## Introduction

The killing of virally infected cells or tumor cells by cytotoxic T lymphocytes (CTLs) is the basis for the adaptive cellular immune response [1]. This process requires the release of cytotoxic substances from lytic granules (LGs) via exocytosis at a CTL-target cell contact area, the immunological or immune synapse (IS) [2]. LGs are considered to be hybrid granules which exhibit the properties of both lysosomes and secretory granules [3–6]. LGs express the lysosomal membrane markers LAMP1 and LAMP2, contain proteolytic lysosomal enzymes and have an acidic lumenal pH [3]. LGs emerge from the endosomal/lysosomal compartment [7], and may result from the fusion of lysosomes with vesicles in which the cytotoxic proteins granzyme A, granzyme B and perforin are stored [8]. Few granules containing granzymes and perforin are observed prior to activation. After activation CTL grow and the number of granules containing lytic components increases rapidly [9,10]. Activated CTLs contain a variety of organelles which could be lytic in nature, differing not only in size but also in appearance, including complex multivesicular bodies containing dense material [3]. It is not known which of these structures represent mature LGs.

The presence of granule markers such as LAMP1 or cytotoxic granule content such as granzymes or perforin do not indicate maturity. These proteins are already present in granules which carry the mannose-6 phosphate receptor, a marker of endosomal compartments [3,4] which is required for the import of granzymes [11]. The mannose-6 phosphate receptor is lost prior to maturation of lysosomes and LGs [12].

During the formation of the IS LGs are actively transported along the microtubuli and the actin cytoskeleton towards the IS. Upon arrival at the plasma membrane, these LGs have to undergo several maturation steps before fusion. First, they are docked at the plasma membrane through the physical interaction of SNARE and SNARE-associated proteins on the vesicle membrane and the plasma membrane, respectively [13]. Second, they are rendered fusion-competent through the action of proteins of the Munc13 family [14], a process called priming [15]. Failure in the docking or priming process of LGs results in lethal immunopathological disorders, Griscelli syndrome type 2 and familial hemophagocytic lymphohistiocytosis type 3, which are caused by mutation in the Rab27a [16] or the Munc13-4 gene [17], respectively. Technically, all plasma membrane-confined steps of LG behavior, i.e. docking, priming and fusion, can be quantified by total internal reflection fluorescence (TIRF) microscopy. In TIRF, docked vesicles display a longer dwell time, since they stay longer at the plasma membrane due to the above-mentioned interactions of vesicle and plasma membrane proteins [18]. Primed vesicles can be distinguished from docked vesicles by a decrease in mobility [19,20]. Finally, fusion of LGs can be visualized in real-time in TIRF microscopy [21]. While real-time analysis of fusion in TIRF yields valuable information about the kinetics of fusion, it cannot be used to extrapolate the size of the fusing vesicle. For this purpose, membrane capacitance measurements are ideally suited. The capacitance of the plasma membrane is directly proportional to its surface area; therefore, every fusing vesicle increases the surface of the plasma membrane by its own surface area. Assuming an increase of 1 μF per cm^2^ surface area for biological membranes [22], the measured capacitance increase upon fusion of single vesicles has been successfully used to estimate the diameter of vesicles in synapses (70 aF; [23]) and neuroendocrine cells (1.3 fF; [24]).

We transfected primary human CTLs with a red fluorescent protein (mCherry) fused to the C-terminus of granzyme B [25] and then carried out TIRF microscopy to identify exocytosis of *bona fide* LGs and to observe granule movement. We simultaneously recorded membrane capacitance [26,27] in the whole-cell mode, which allowed us to identify membrane capacitance steps associated with LG exocytosis. LG capacitance steps indicate a population of granules with an estimated diameter of ~ 325 nm. LG fusion occurred with a mean latency of 55 seconds following introduction of ~ 2 μM intracellular free [Ca^2+^] via the pipette solution rupture. This is the first report of whole-cell capacitance recordings from primary CTLs and allowed measurement of single granule fusion events with high resolution.

## Materials and Methods

### Human cytotoxic T lymphocyte preparation and electroporation

Experiments were performed on human CTLs in primary culture. Human peripheral blood lymphocytes were obtained from healthy donors as described previously[28]. Naïve CTLs were negatively isolated using a Dynabeads Untouched human CD8 T-cell kit (Life technologies) and stimulated with Dynabeads Human T-Activator CD3/CD28 (Life technologies) for 2 days. At day 3, 5 x 10^6^ CTLs were transfected with the granzyme B-mCherry construct (see below) using electroporation (Human T cell Nucleofector kit, Lonza, Germany). Transfected CTLs were used 12–16 hours after transfection.

### Ethics Statement

Experiments on CTLs were performed with leucocytes provided by the Saarland University Medical Center Hematology Department blood bank (http://www.uniklinikum-saarland.de/de/einrichtungen/kliniken_institute/chirurgie/haemostaseologie/). Written informed consent of the donors was obtained. Donor information was anonymized. Approval for use of the cells for research was obtained from the Ethics Commission of the Saarland University medical center.

### Human granzyme B-mCherry cDNA construct

Granzyme B was amplified from human cDNA with primers: 5’-TAT ACT CGA GCC ACC ATG CAA CCA ATC CTG CTT CTG-3’ and 5’-ATA TAT CCG CGG GTA GCG TTT CAT GGT TTT CTT T-3’ to add XhoI and SacII restriction sites at each end. The mCherry construct was a gift from Prof. Roger Tsien. After digestion with XhoI and SacII, granzyme B was ligated to vectors containing mCherry-N1 at the C-terminal end of granzyme B and then purified.

### TIRF microscopy

All experiments were done using an Axiovert 200 microscope (Zeiss, Göttingen, Germany) equipped with a Zeiss TIRF-slider and a solid-state laser (85YCA010; Melles Griot, Carlsbad, CA) emitting at 561 nm. To observe mCherry fluorescence (excitation maximum ~ 590 nm; [29]), a filter set containing an UV-reflecting dual-band dichroic mirror (catalog #F53–563; AHF Analysentechnik, Tübingen, Germany) and an emitter (catalog #F72–419) were installed. We used a 100x/1.45 NA Fluar objective (Zeiss) for all measurements. Images were acquired using an EMCCD camera (Andor iXonEM, Belfast, Ireland) and controlled by software written in house in the LabView environment (National Instruments, München, Germany). Pixels were 160 x 160 nm. Images were acquired at 10 Hz with an exposure time of 75 msec. Tracking of LGs was done using a centroid algorithm with software written in house [18]. Frame to frame movement was calculated as the square root of the sum of the squares of the X and Y displacements. This software was also used to calculate caging diameter.

### Granule fusion determination

Videos of TIRF images were analyzed using Image J (NIH, USA). Fusion resulted in a rapid decrease in LG brightness and the released fluorescent granzyme B produced a cloud of fluorescence which rapidly dissipated. Most TIRF fusion events exhibited both a loss of granule fluorescence and a local cloud of fluorescence, though in some cases a cloud appeared without a visible granule. The latter observation may result from dimpling of the membrane taking the granule out of the TIRF field. The TIRF video acquisition was continuous and began prior to the capacitance record.

### Whole-cell patch-clamp recordings

Glass coverslips (25 mm) were treated with 100 μl poly-L-ornithine (Sigma, USA) for 30 minutes at room temperature. A small drop of a mixture of anti-CD3 (30 μg/ml) and anti-CD28 (90 μg/ml) antibody was then applied to the coverslip, spread over its surface and the coverslips were incubated at 37°C for two hours. Suspensions (60 μl) of activated CTLs were plated onto these coverslips in a HEPES-buffered, nominally calcium-free physiological salt solution (in mM, NaCl; 155.0, MgCl_2_; 3.0_,_ KCl; 4.5, HEPES; 5.0, pH = 7.4) and allowed to settle for 5 minutes. The coverslip was then placed in a recording chamber on the stage of the microscope and cells were recorded at room temperature. Cells that are in contact with the anti-CD3/anti-CD28 antibodies adhere and change shape, going from mobile, flat cells to rounded, immobile cells during the activation process. T cell receptor (TCR) binding to the anti-CD3 antibody leads to clustering which is required for activation [30].

Patch-clamp recordings were carried out in the whole-cell configuration. Patch pipettes were pulled from thick-walled borosilicate glass (GB150F-8P, Science Products, Germany). The pipette resistance was 2.5–3.5 MΩ. Sigmacote (Sigma, USA) was applied to the pipette tips. The pipette solution contained (in mM): glutamic acid, 100; HEPES, 40; Cs-EGTA, 5.9; CaCl_2_, 5.4; Na_2_GTP, 0.3; MgATP, 2.0. The pH was adjusted to 7.3 with CsOH and the osmolarity was ~ 307 mOsm.

A CTL was located and centered in the TIRF field. The footprint was brought into focus and the illumination angle adjusted to achieve TIRF. The cell was patch-clamped if mCherry-fluorescent labeled LGs were present at the CTL-coverslip interface. After a GΩ seal was formed, the focus was adjusted and TIRF-video acquisition begun. After a short TIRF recording period, suction was applied to the pipette, rupturing the patch membrane to attain a whole-cell recording.

The capacitance was compensated and the capacitance record acquisition started. Cells were clamped at a holding potential of -70 mV using an EPC-9 patch-clamp amplifier controlled with the “lock-in” extension of PULSE software (Heka, Germany). The sine + DC method was used with a sine wave frequency of 1000 Hz and amplitude of 50 mV RMS. The voltage-clamp data were acquired as a single continuous record. The camera trigger signal was recorded to allow correlation of video with capacitance data.

Fusion events produce discrete jumps in membrane capacitance [27]. In order to improve accuracy of measurement of the amplitude of capacitance steps, the capacitance traces were filtered using a low pass filter implemented in Igor Pro 6 (Wavemetrics, USA). This allowed us to distinguish steps smaller than 1 fF. The accompanying membrane conductance and access conductance measurements were also recorded. Stepwise increases in capacitance were discarded if they were accompanied by abrupt changes in either the access- or membrane conductance, since such events, in particular those in the access conductance trace, can cause a change in the capacitance trace [31]. We averaged the 60 points prior to the step and the 60 points after the step and took the difference as the amplitude of the capacitance step.

### Statistics

Statistical calculations, curve fits, the Kolmogorov-Smirnov test and the Mann-Whitney U test were implemented in Igor Pro.

## Results

### Membrane capacitance measurements of human cytotoxic T lymphocytes

We carried out membrane capacitance recordings in the whole-cell mode [32] from human CTLs in primary culture. Though a number of cell lines have been used for patch clamp experiments of lymphocytes there have been few reports of properties of primary CTLs, due in no small part to the difficulty of recording these cells, and no examination of LG exocytosis using capacitance measurements. Capacitance measurements combined with TIRF microscopy were carried out in 38 CTLs to identify the capacitance steps associated with LG fusion.

**Fig 1:**
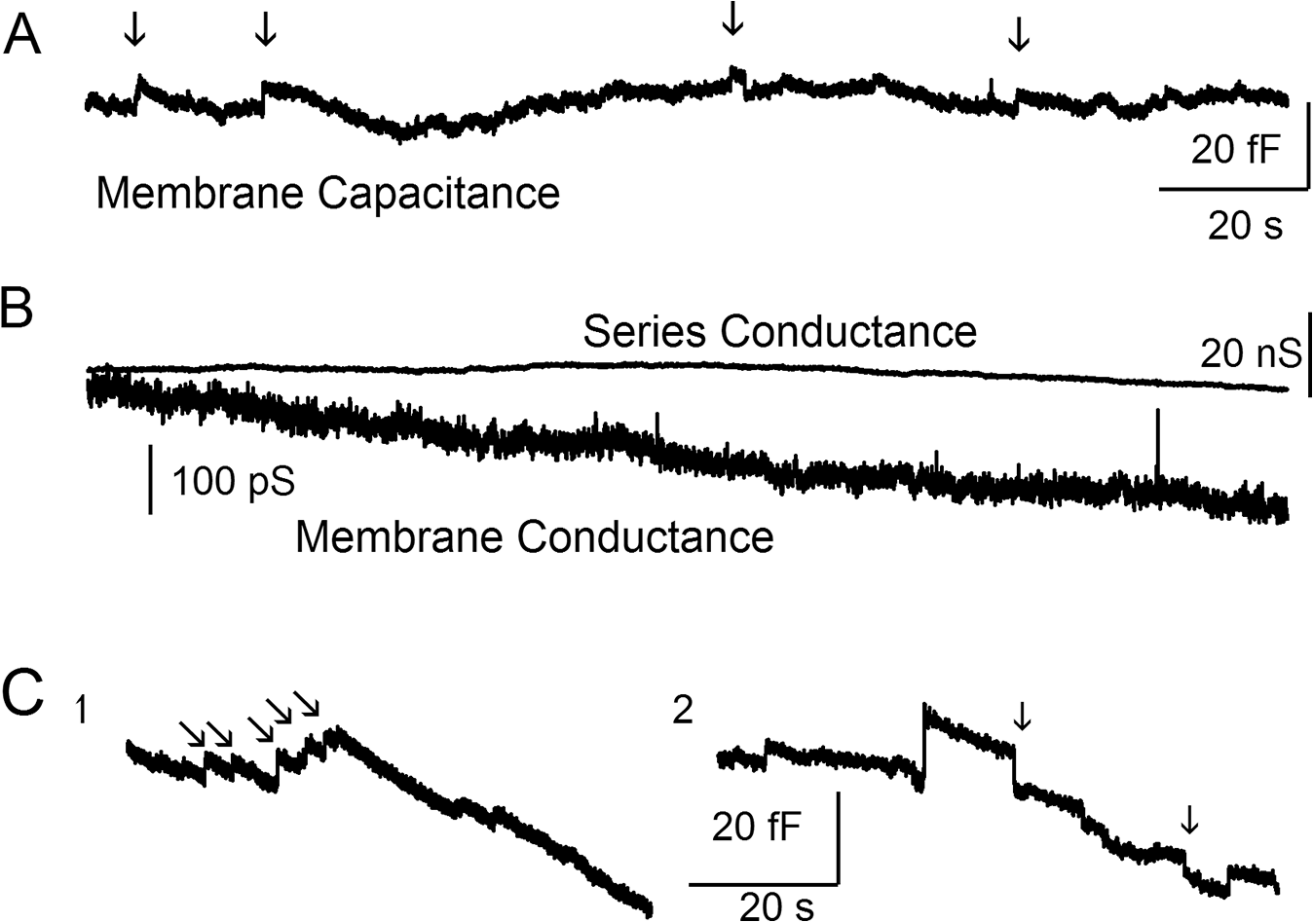
Capacitance steps in stimulated CTLs. (*A*) Examples of step capacitance responses in CTLs. The arrows indicate steps associated with granzyme B positive exocytotic events in TIRF images. (*B*) The series conductance and the membrane conductance are shown (*C*) Examples of a cluster of positive steps indicating exocytotic events (part 1) and of negative steps (part 2, downward) which indicate endocytosis (arrows).

Rupture of the patch membrane to achieve the whole-cell configuration was often followed by a rapid increase in membrane and access conductance that stabilized within 10 to 20 seconds. Small, rapid positive steps in the whole-cell capacitance trace were observed at a low frequency (at peak secretion the inter-event intervals were rarely less than 500 ms) in all recorded cells. Representative traces are shown in Fig 1. The membrane conductance and the series conductance traces are shown to verify that steps in the capacitance trace are not artifacts resulting from changes in recording quality (see Materials and Methods). Six hundred and six capacitance steps with mean amplitude of 3.98 ± 2.65 fF (mean ± s.d.) were recorded.

### Combined membrane capacitance measurements and total internal reflection fluorescence microscopy in human cytotoxic T lymphocytes

**Fig 2:**
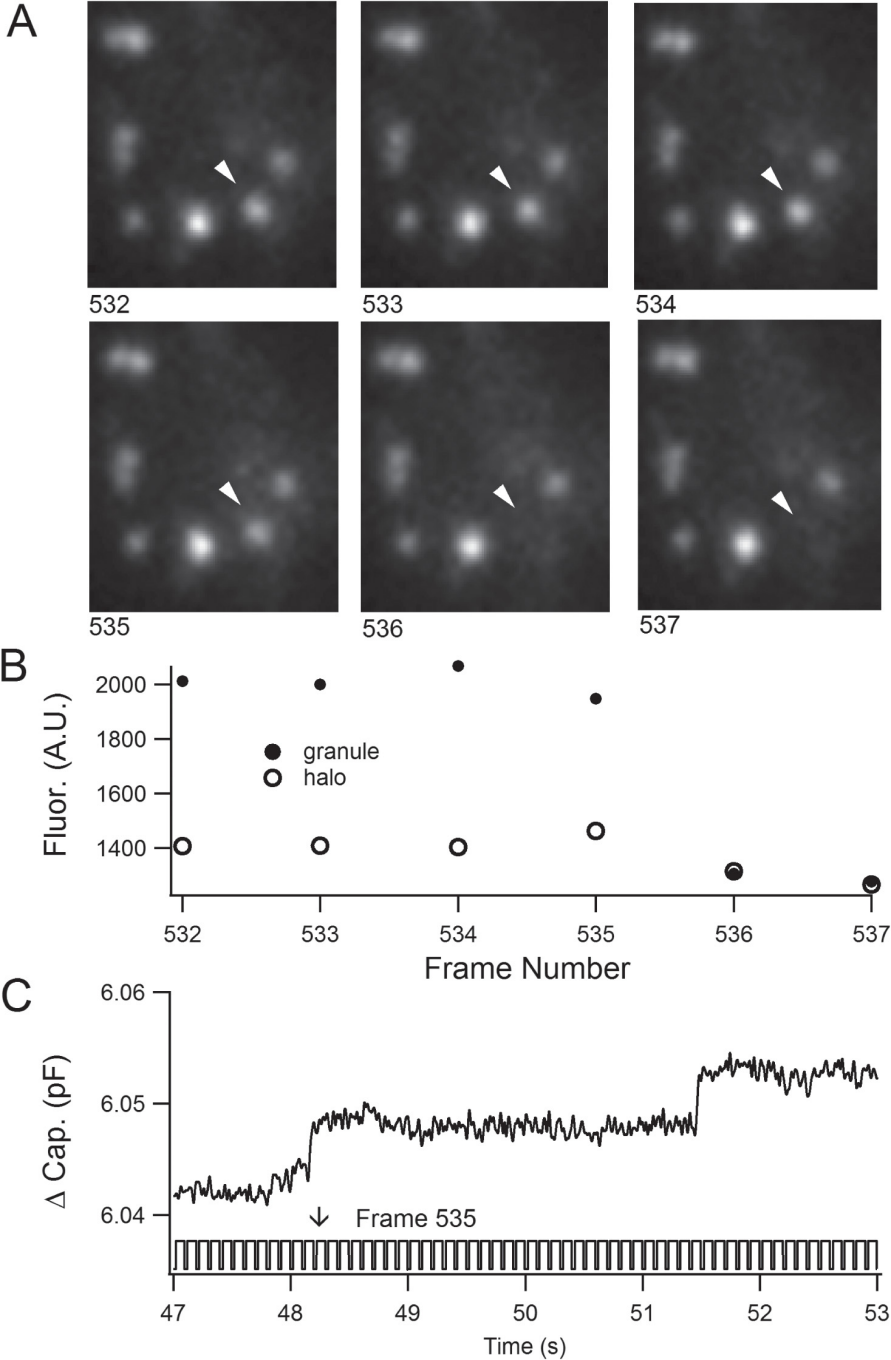
Fusion of a granzyme B-mCherry containing lytic granule with the plasma membrane. (*A*) Consecutive frames from the TIRF video imaging are shown. The video frame numbers are shown below the images. The arrowhead indicates a lytic granule which decreased in fluorescence and generated a cloud of fluorescence while fusing. (*B*) The fluorescent intensity (arbitrary units) of the fusing granule (filled circles) indicated by the arrow in *A* was plotted versus frame number. The intensity of the juxtagranular region (halo, open circles) is included. As the granule fused its intensity increased briefly (frame 534) and it then disappeared. At frame 535 the juxtagranular area brightened. In subsequent frames the fluorescence dissipated (frame 537). (*C*) The whole-cell membrane capacitance trace (C_m_, upper trace) and the camera trigger voltage are shown (lower trace, positive steps indicate shutter opening) versus time. The capacitance step in the C_m_ trace at time 48.2 s occurs at video frame 535 (arrow).

Use of TIRF microscopy to identify LG fusion events in CTL induced to form an IS at a treated cover slip is well established [25,33,34]. Granzyme B was chosen as the LG marker because it is present in human CTLs and its expression increases upon activation [35]. Seeding of CTLs on anti-CD3/anti-CD28 coated coverslips results in microtubule reorientation, accumulation of actin, CD3, and lytic granules at the CTL-coverslip interface, and LG release consistent with IS formation [21]. Since a stable increase in intracellular [Ca^2+^] is required for effector function in CTLs [36,37], we supplied ~ 2 μM free intracellular Ca^2+^ via the patch pipette. The experiments were carried out in a nominally calcium-free extracellular solution to slow CTL activation and to prevent the release of granules prior to recording. We observed LG fusion in 21 of 38 recorded CTLs perfused with ~ 2 μM free intracellular Ca^2+^. In cells patched with a free intracellular [Ca^2+^] below 280 nM, no fusion events could be observed (data not shown), demonstrating the [Ca^2+^] dependence of LG fusion. A typical TIRF fusion event is shown in Fig 2A. Loss of LG fluorescence accompanied by a cloud of fluorescence in the juxtagranular area was considered an indicator of LG fusion (Fig 2B, S1 Video). Fusion can be distinguished from movement of the granule from the plasma membrane by the cloud and by the rapidity of the fluorescence loss (a single frame versus an incremental decrease in brightness over several frames). LG fusion events occurred sporadically and in CTLs in which LG release occurred, there were 5.02 ± 3.09 (mean ± s.d.) LG fusion events per cell.

In frames in which fusion occurred, there were 9.39 ± 3.98 (mean ± s.d.) LGs present. CTL never depleted their stock of LGs and LGs sometimes arrived at the membrane late in the recordings when release had already ended. Only a fraction of LGs fused. This was expected since a small number of LGs is sufficient for killing and CTLs can kill multiple target cells [38]. A similar result was noted in killing assays carried out on Tall 104 cells (stable cell line established from peripheral blood of a child with acute lymphoblastic leukemia) stimulated by application of thapsigargin and phorbol-myristoyl-acetate (which bypasses the need for TCR stimulation) [39]. The mechanisms restricting numbers of fusing granules is of great interest. Since release also appears to be limited in our experiments, apparently there is not a requirement for a signal from the target cell. No fusion events were observed in the period between establishing TIRF illumination and membrane break-in to the whole-cell mode (~10–20 seconds).

The simultaneous capacitance recording (Fig 2C) shows a step-like increase at 48.2 seconds. The lower trace in part C shows the camera trigger signal. Frame 535 from the video (panel A) is indicated (↓) and is the frame prior to fusion (loss of fluorescence) in TIRF. Following identification of LG fusion in TIRF, we examined the capacitance traces for candidate capacitance steps. Capacitance steps that began prior to or during the first TIRF frame showing fusion and which were tightly coupled in time with TIRF fusion were considered to be LG release events (see Fig 2). Forty-seven of the ninety-eight capacitance steps that correlated with TIRF events occurred during the video frame in which a fusion event began. Three fusion events in TIRF could not be assigned unambiguously to a capacitance step.

Using the drawing tool of ImageJ we marked the area in which granule fluorescence was visible in TIRF, indicating close contact between CTL and coverslip, at any time during the recording. We then compared the area of this TIRF “footprint”, which is smaller than the contact area of the cell with the coverslip (measured with fluo-4), to the surface area of the CTL calculated from its whole-cell capacitance. The average footprint encompassed 12.1 ± 8.6% (mean ± s.d.) of the CTL surface area and had a mean area of 103 μm^2^ which is sufficient for an annular contact zone with a diameter of 10.2 ± 3.2 (mean ± s.d.) μm. The dimensions are consistent with CTL-target cell immune synapses observed in live cell imaging [40]. The number of fusing LGs was independent of CTL size, footprint size and the ratio of size of the footprint to CTL size (see S1 Fig).

In cells in which fusion was observed in TIRF, 26% of the capacitance steps (98/371) were correlated with an LG fusion event. Since membrane capacitance measurements report the increase in surface area of the entire cell, the remaining 74% of the capacitance steps were therefore fusion events of non-fluorescent vesicles, i.e. not lytic granules, inside and outside of the IS. It was shown, for example, that recycling endosomes frequently fuse with the plasma membrane to deliver cargo like the TCR and syntaxin11 [25,41] In light of the relatively small area of the cell involved in the footprint (12.1%, see above) the observation that 26% of the observed capacitance steps were identified as LG fusion in TIRF indicates that LG fusion occurred preferentially at the footprint as expected for an IS. If this were not the case, LG fusion alone would account for 834 steps.

### Lytic granules fusing with the plasma membrane have a diameter of 325 nm

**Fig 3:**
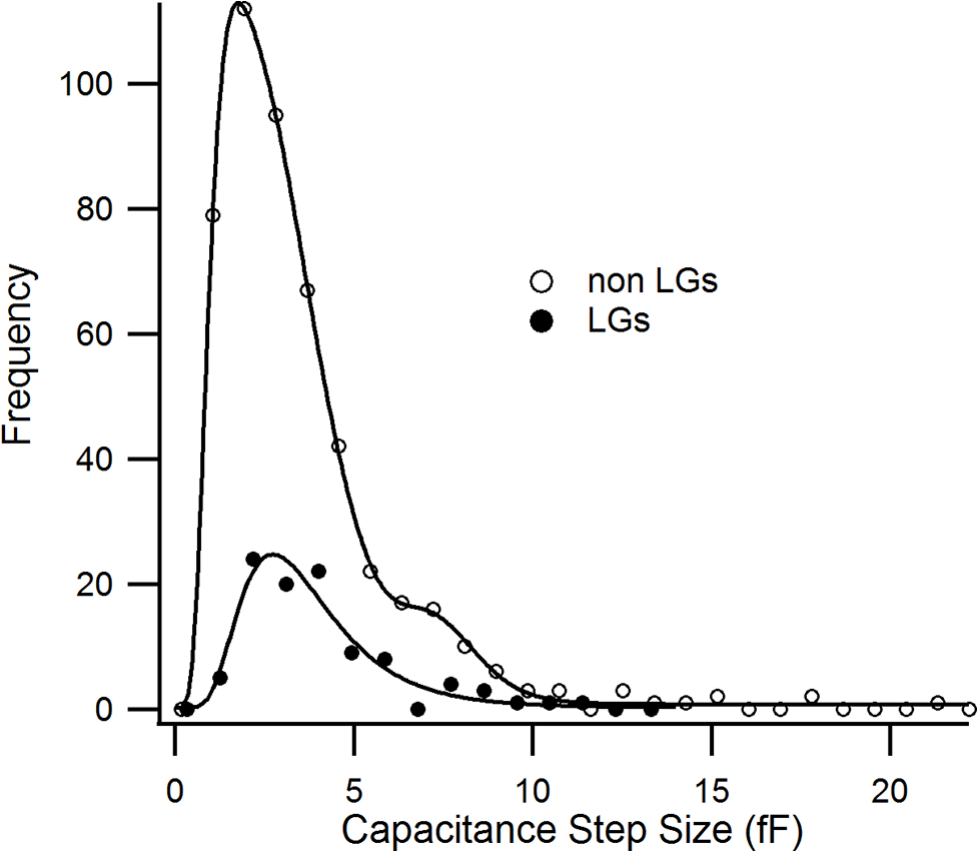
Capacitance steps associated with lytic granule fusion events indicate a population with a diameter near 325 nm. The frequency histograms of capacitance steps observed in CTLs. The open circles show the frequency histogram of 508 fusion events obtained from 38 CTLs which were not related to LG fusion. The histogram is fit with a triple Log-normal equation (solid line). The filled circles show the histogram of 98 LG fusion events, which is fit with a single Log-normal (solid line).

Fig 3 shows frequency histograms for all observed capacitance steps and those associated with LG fusion (see also S1 Table). The frequency histogram for LG capacitance events indicates few very small (less than 1.4 fF) capacitance steps. The histogram for non-LG events was fit using a triple lognormal fit with peaks at 2.2, 3.3 and 8.2 fF. The frequency histogram of LG capacitance steps was fit with a single lognormal curve with a peak at 3.3 fF. Since it was shown that fusogenic LGs are spherical [42], this capacitance value translates to a granule diameter of 325 nm, assuming an increase of 1 μF per cm^2^ surface area of biological membrane [21]. Our cutoff of 1 fF may result in an underestimate of non-lytic granules but very few LGs approached this cutoff so our estimate of LG size is not affected.

The amplitudes of LG steps and non-LG steps were significantly different (p ≤ 0.05, Mann-Whitney U test). Our estimate of LG size is consistent with a report of a homogeneous population of granules obtained from fractionated T cells following high-density Percoll gradient centrifugation [43]. The granules contained lysosomal enzyme activity, lacked markers for rough endoplasmic reticulum and Golgi compartments, were LAMP1- and β-glucuronidase-positive, but lacked the mannose-6 phosphate receptor. The authors concluded that these granules were mature LGs. Based on their EM images with immuno-gold labeling, those granules were near 320 nm in diameter which is in excellent agreement with our estimate based on capacitance.

### Fusing lytic granules exhibit long dwell times

**Fig 4:**
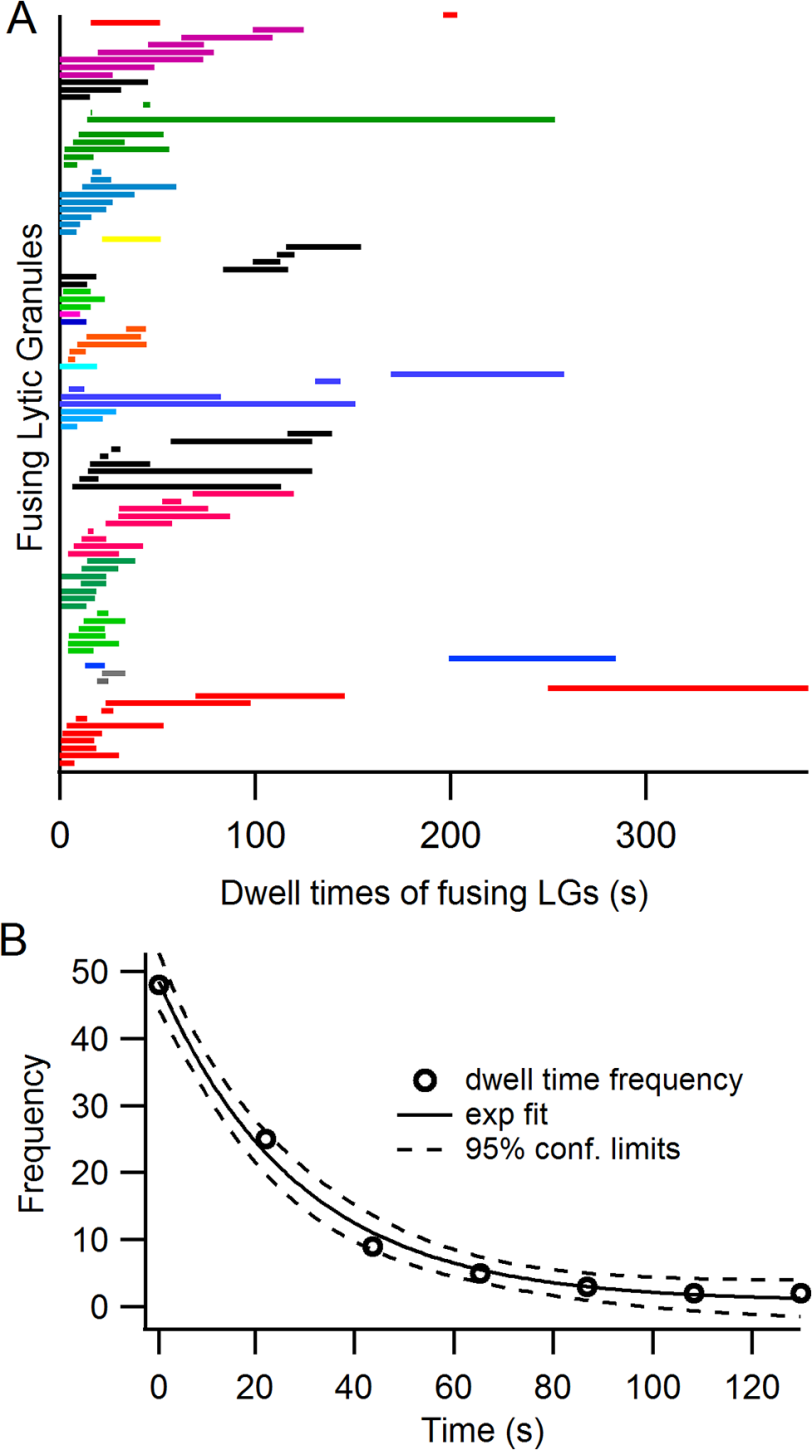
Lytic granules which undergo fusion can exhibit long dwell times. (*A*) The dwell times of 101 LGs which fused are shown. Each LG is represented as a horizontal bar beginning at the start of its sojourn at the membrane and ending at its fusion. Contiguous bars with the same color indicate granules from the same CTL. Most fusing granules appear at the membrane early and most granules which fused exhibited a relatively short dwell time. (*B*) The frequency histogram of dwell times could be fit with a single exponential distribution (solid line, 95% confidence limits are indicated by the dashed lines) with a mean near 28.5 seconds.

After arriving at the immune synapse LGs must dock and prime before fusing [2]. Docking involves an interaction between SNARE proteins located on LGs with SNARE proteins on the plasma membrane [44]. SNARE complex formation will result in a decrease in the freedom of motion of the LG and is reflected in increased dwell time, the duration of the time a granule resides continuously at the plasma membrane, and in a decrease in mobility of LGs. We determined LG dwell time in the TIRF field (Fig 4A). Thirty-one of the 101 fusing LGs were in the TIRF field prior to break-in to the whole-cell mode. The dwell times ranged from under a second (n = 3) to 239 seconds (Fig 4A). Thus, LGs (whether they fuse or not) can exhibit dwell times of tens of seconds or minutes. The distribution of dwell times of fusing LGs (Fig 4B) was well fit with an exponential with a mean dwell time of 28.5 seconds (see also S2 Table).

### Lytic granules at the immune synapse show different types of movements

**Fig 5:**
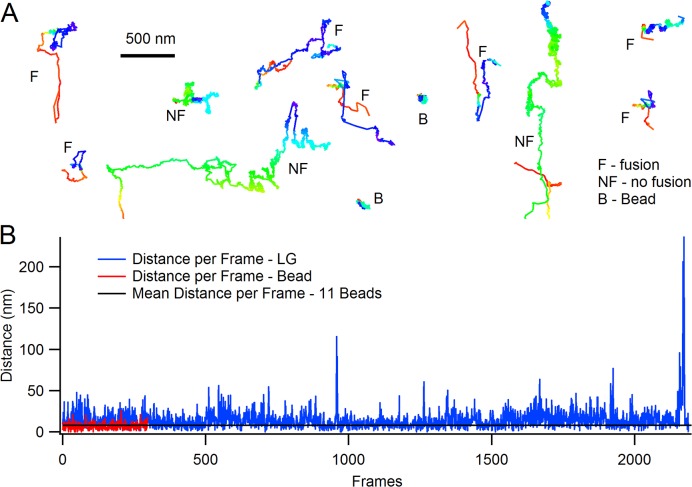
The behavior of lytic granules at the CTL-coverslip interface. (*A*) Representative tracks of granules in a single CTL are shown. Seven tracks (F) indicate LGs which underwent exocytosis (blue is the track end). Three tracks (NF) are LGs which did not fuse. The tracks are to scale but have been rearranged to decrease the size of the image and improve visibility. Two representative tracks from fixed beads (B) which were recorded prior to the CTL experiment are included to show intrinsic jitter of the workstation. (*B*) A plot of the frame-to-frame movement of a representative track. The red trace is a representative track from a fixed bead. The mean movement of 11 fixed fluorescent beads (0.2 μm in diameter, black trace) is superimposed on the plot.

The prolonged dwell times may indicate that LGs dock at the plasma membrane. We examined the mobility of LGs dwelling at the membrane and found that they exhibit multiple periods of very low mobility consistent with granule docking [45,46] interspersed with short periods of higher mobility. Representative tracks are shown in Fig 5A. Seven LGs which fused and three LGs which did not fuse are shown. All ten tracks are from the same CTL. The tracks of two fluorescent beads (0.2 μM diameter) which were fixed to coverslips are included in the panel (these were not recorded during the LG recordings). The beads are included to show intrinsic jitter of the recording system. The frame-to-frame movement of the fixed beads was quite constant and ranged from 6–12 nm (8 ± 2, mean ± s.d.) for eleven beads (see S3 Table).

The distance moved per frame for a representative track of an LG is shown in Fig 5B. Also shown are a representative track from a fixed bead and the average movement per frame for 11 fixed beads. The distances moved per frame in segments of the LG track approached that of fixed beads during its residence in the TIRF field.

Thus, though the tracks are variable, LGs move in the TIRF field sporadically, and can remain in an almost immobile state approaching that of the fixed beads, consistent with docking [47,48] for tens of seconds without fusing, and then move further. This behavior is typical. Limited mobility of LGs in the TIRF field may depend on a cortical actin network which regulates or limits access of granules to the plasma membrane [45,49,50], but an additional loss of mobility is expected when granules are tethered and eventually dock [47,48].

### Fusing lytic granules cannot be distinguished from non-fusing lytic granules by their mobility

**Fig 6:**
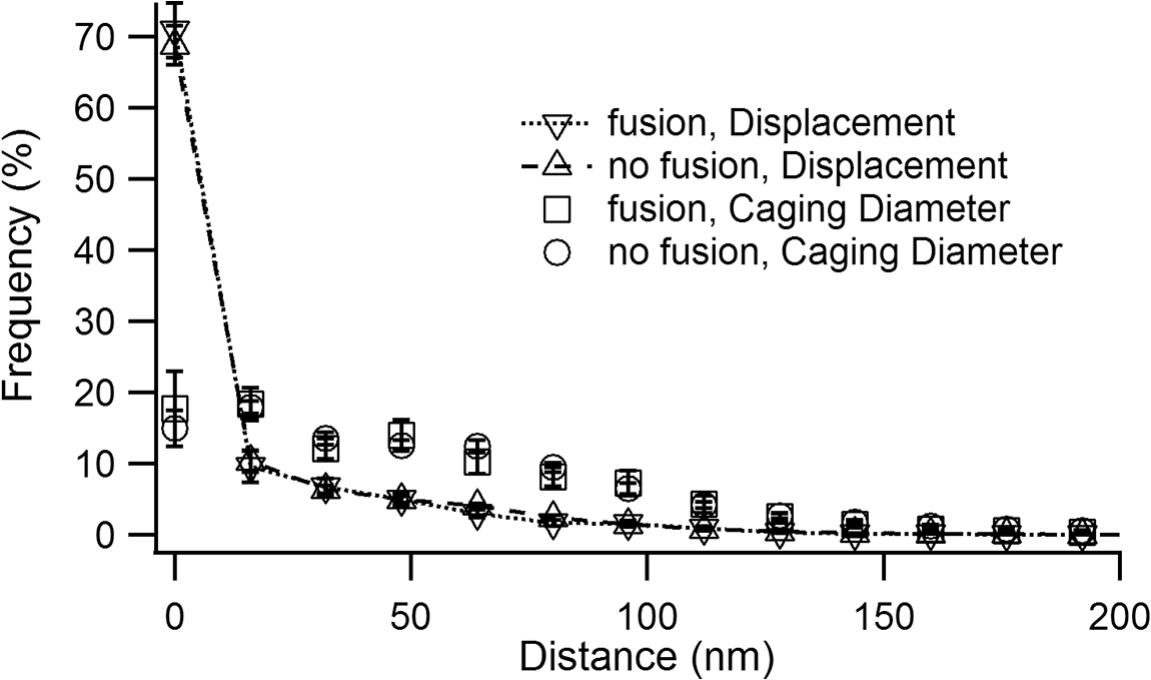
Fusing and non-fusing LGs cannot be distinguished based on their mobility. (*A*) The averaged frequency histograms of LG displacements are shown.The frame to frame displacement of the tracks of 58 granules which fused and 816 granules which did not fuse were calculated. There was no difference between the distributions of displacements of fusing LGs and those which did not fuse. (*B*) The caging diameters (see text) were calculated and the averaged frequency histograms of the largest distances covered in ten frames (1 second) are shown for fusing and non-fusing LGs. There was also no difference between the caging diameters of fusing and non-fusing LG.

In neuroendocrine cells it was shown that docked and primed large dense core vesicles can be distinguished based on their mobility [19]. We compared the mobility of LGs which fused to that of those which did not fuse. We calculated the displacement of LGs (see Materials and Methods) and then compared the frequency distribution of the frame-to-frame displacement of LGs which fused to that of granules which did not fuse (Fig 6). The distributions of both groups, fusing and non-fusing LGs, were strongly skewed toward small distances consistent with the low mobility mentioned previously, and were not significantly different. Non-fusing LGs often exhibited long dwell times and some granules were present for the entire recording. In spite of the preponderance of small displacement values, granules could either move laterally or remain more or less in place which results in quite different net movement. To differentiate these two conditions we determined a caging diameter [19] which is the greatest distance reached between an LG position and its position in the following N frames. This analysis was carried out for all frames using a ten frame (1 s) sliding window. The frequency histograms of the caging diameters for 58 tracks of fusing granules and 812 tracks of non-fusing granules (the same granules used for displacements) are also shown in Fig 6 (see S4 Table). There was no difference between the frequency distributions of caging diameter for fusing and non-fusing granules. Since fusing LGs must dock at the membrane and are by definition mature, the inability to distinguish between the behavior of fusing and non-fusing granules supports the conclusion that non-fusing granules are also mature and that failure to fuse is due to a lack of a membrane-confined reaction. The lack of a difference between LGs which fuse and those which do not fuse is likely due to the immediate fusion of LGs which reach the primed state, precluding observation of primed LGs. This is expected since the free intracellular [Ca^2+^] is more than sufficient to trigger fusion.

### Fusion of lytic granules is biphasic

**Fig 7:**
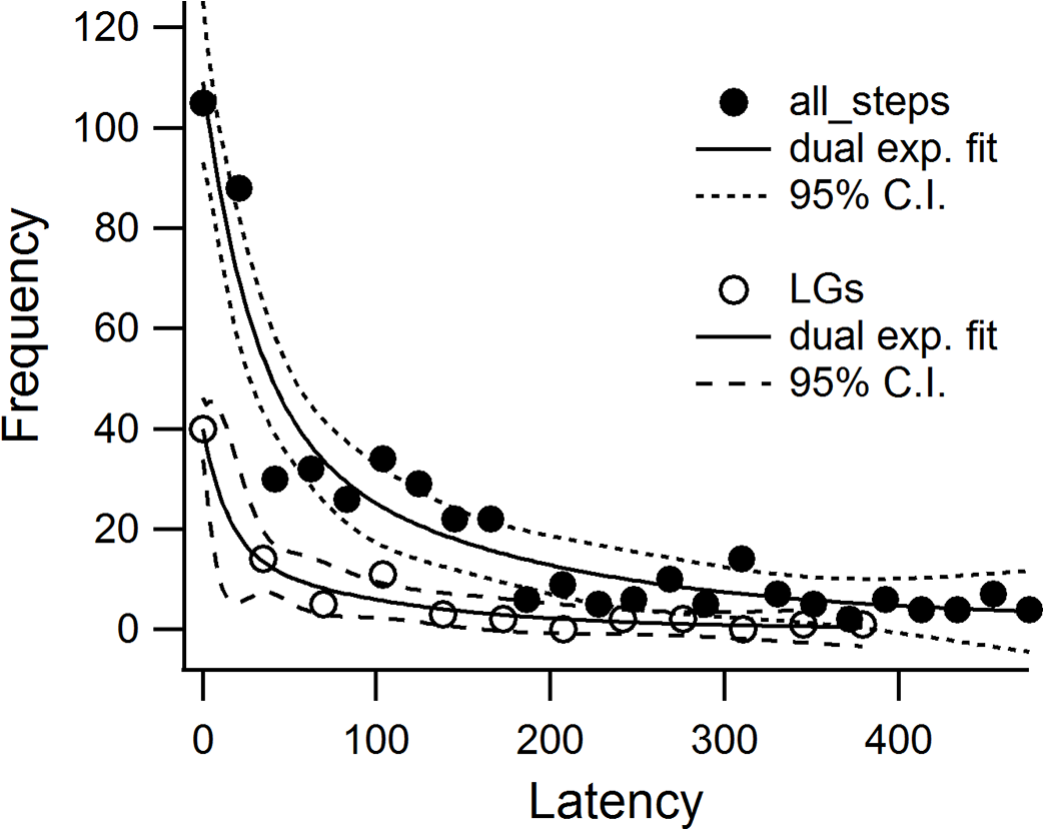
Fusion of LGs is biphasic. Frequency histograms show the latency from calcium perfusion to fusion in CTLs. The filled circles represent the frequency histogram for all capacitance steps. These were fit with a double exponential (solid line) with a fast time constant of 28.2 s and a slower time constant of 148.4 s. The open circles represent the frequency histogram of the capacitance steps associated with LG fusion. This histogram was well fit with a dual exponential (solid line) with a fast time constant of 15.9 s and a slow time constant of 116.3 s. The 95% confidence intervals are indicated by dashed lines in both curves.

The latency to fusion, the interval between beginning calcium perfusion and LG fusion, will depend on the status of LGs. Primed LGs should have a short latency to fusion since they only require an increase in intracellular free calcium. LG fusion events occurred with an average latency of 55 seconds. The distribution was skewed to shorter times, with few events having a latency of more than 120 seconds. Frequency histograms of capacitance-step latency after break-in for LGs versus all steps are shown in Fig 7. The frequency histogram for all events was fit with a dual exponential with time constants of 28 and 148 seconds. The latencies of LGs were significantly different (p ≤ 0.01, Mann-Whitney U test) from those of non-LGs. The latency to release of LGs was biphasic with a rapid (15.9 s) and a slower (116.3 s) phase (Fig 7, see also S5 Table). The rapid phase may include a small fraction of LGs which are primed at the time of synapse formation and require only an increase in calcium for fusion, but the latency of 15.9 seconds likely cannot be explained simply by diffusion of calcium after patch rupture. This idea is supported by the very short delay observed for some granules, and thus likely reflects delayed priming. The second component may reflect granules which have a lower priming rate, perhaps due to limited availability of priming factors.

The fast time constant (28 seconds) observed in the latency histograms for all events includes the fast phase of LG release since the time constant of rapid LG release fell in this interval. It was expected that LG release would be limited and that non-LG fusion events occur and continue throughout the recording, since membrane trafficking in CTLs is involved not only in delivery of LGs, but also in adherence, IS construction, delivery of proteins and membrane to the IS and in motility [51–54].

## Discussion

Combined patch-clamp recording and TIRF microscopy allowed us to identify capacitance steps associated with fusion of mature LGs in human CTLs. This allowed us not only to estimate LG size at the time of fusion, but also to examine their mobility prior to fusion.

Our results demonstrate that LG fusion accounts for about 1/4 of the capacitance steps observed. On average, secreting CTLs had five LG fusion events. Considering that the content of one or few LG is sufficient to mediate efficient target cell killing *in vivo*, the obtained LG fusion number per cell is rather high. Most likely, this is due to the high intracellular Ca^2+^ (~ 2 μM) applied via the patch pipette which we used to maximize LG fusion in our challenging experimental paradigm. In principle, the remaining 74% of the measured capacitance steps could originate from two sources. The first possible source would be LGs which fuse outside of the visualized evanescent field. We consider this scenario highly unlikely, because time-lapse confocal microscopy of LGs transfected with the same construct as used here (granzyme B-mCherry) revealed that all LGs polarize to the IS upon target cell contact within a few minutes (data not shown). Since we induce a reliable IS at the cell-coverslip interface through anti-CD3/anti-CD28 coating, we are confident that all LGs polarize to this IS and become visible in the evanescent field. The second possible source is the fusion of other, intracellular organelles like recycling endosomes, lysosomes, mitochondria etc. (i.e. non-LGs). These non-LGs could fuse outside the evanescent field or could fuse at the IS (in the evanescent field), but would not be visualized because they are not fluorescently labeled. Actually, we have provided evidence that recycling endosomes fuse at the IS prior to LG fusion in order to deliver cargo that is required for IS formation [25,41,55]. Since recycling endosomes are smaller in diameter than LGs, we speculate that the measured peak at 2.2 fF (Fig 3) is caused by these recycling endosomes. In addition, activated CTLs show a large increase in cell diameter compared to naïve CTLs due to an increase in transcription, translation and incorporation of proteins into the plasma membrane. Therefore, we conclude that the remaining capacitance steps are most likely caused by this source.

We further determined that the average size of LGs is 325 nm. This size determination is based on two assumptions: a) the membrane of LGs has a specific capacitance of 1 μF per cm^2^ surface area [21]. b) LGs are spherical. While the first assumption is difficult to prove without knowing the exact lipid composition of LG membrane, there is good evidence that the second assumption holds true. First, elegant immuno-gold electron microscopy studies have demonstrated that LGs and lysosomes have a diameter between 300 and 700 nm [4,56]. Second, correlative light and electron microscopy (CLEM) studies with a specific marker for mature, fusogenic LGs, synaptobrevin2, revealed a diameter of 300 nm for LGs [42]. In both cases, LGs appeared as an almost perfect, electron-dense sphere. Thus, it appears that capacitance recordings yield a reliable value for LG size, as it does for other granules, e.g. synaptic vesicles and chromaffin granules [22,23].

Interestingly, our data show that mobility of non-fusing LGs is indistinguishable from that of LGs (Fig 6). This is in contrast to large dense-core vesicles in adrenal chromaffin cells, in which primed vesicles have a considerable lower mobility than docked, but unprimed vesicles [18]. In both systems, CTLs and chromaffin cells, it was shown that granules dock and prime at the plasma membrane upon arrival [57]. However, while chromaffin granules reside in the primed state in releasable pools awaiting Ca^2+^ influx, it is entirely unclear whether LGs spend considerable time in the docked and primed pools before they fuse with the plasma membrane. In addition, the contribution of cytoskeletal components like microtubules or the actin network to vesicle movement is currently unknown.

The establishment of simultaneous patch-clamp and TIRF recordings described in this study will offer possibilities for future studies of LG function. For example, this method will allow the investigation of the docking, priming and fusion of LGs under conditions of controlled intracellular calcium. Furthermore, access to the intracellular space through the patch pipette enables the introduction of specific blockers for the individual steps of granule maturation at the plasma membrane, with the potential to unravel the molecular mechanisms leading to target cell killing through LG release.

## Supporting Information

S1 Fig The relationship between LG release, cell size and footprint size and relative footprint size. The number of released granules is plotted vs these three variables.
(TIF)

S1 Table Fig 3 Capacitance Steps: All steps and LG steps. (TXT)

S2 Table Fig 4 Dwell times: All dwell times and Dwell begin- and end-times for each fusing LG grouped by cell. (TXT)

S3 Table Fig 5A X and Y axis data for Tracks. (TXT)

S4 Table Fig 6 data: MSD and Caging diameter for fusing and non-fusing LGs. (TXT)

S5 Table Fig 7 data for Fusion latency. (TXT)

S1 Video LG fusion in TIRF microscopy. A 17 second long video (avi format) containing 4 fusion events. The video begins 15 s after break in, and is cropped to an area 8.3 μm (X-axis) by 7 μm (Y-axis, the horizontal bar is 5 μm long).
(AVI)

## References

[1] Zhang N, Bevan MJ. CD8(+) T cells: foot soldiers of the immune system. Immunity. 2011;35: 161–168. 10.1016/j.immuni.2011.07.010 21867926 PMC3303224

[2] de Saint Basile G, Ménasché G, Fischer A. Molecular mechanisms of biogenesis and exocytosis of cytotoxic granules. Nat Rev Immunol. 2010;10: 568–579. 10.1038/nri2803 20634814

[3] Burkhardt JK, Hester S, Lapham CK, Argon Y. The lytic granules of natural killer cells are dual-function organelles combining secretory and pre-lysosomal compartments. J Cell Biol. 1990;111: 2327–2340. 2277062 10.1083/jcb.111.6.2327 PMC2116378

[4] Peters PJ, Borst J, Oorschot V, Fukuda M, Krähenbühl O, Tschopp J, et al Cytotoxic T lymphocyte granules are secretory lysosomes, containing both perforin and granzymes. J Exp Med. 1991;173: 1099–1109. 2022921 10.1084/jem.173.5.1099 PMC2118839

[5] Bossi G, Trambas C, Booth S, Clark R, Stinchcombe J, Griffiths GM. The secretory synapse: the secrets of a serial killer. Immunol Rev. 2002;189: 152–160. 12445272 10.1034/j.1600-065x.2002.18913.x

[6] Marks MS, Heijnen HFG, Raposo G. Lysosome-related organelles: unusual compartments become mainstream. Curr Opin Cell Biol. 2013;25: 495–505. 10.1016/j.ceb.2013.04.008 23726022 PMC3729921

[7] Page LJ, Darmon AJ, Uellner R, Griffiths GM. L is for lytic granules: lysosomes that kill. Biochim Biophys Acta. 1998;1401: 146–156. 9531970 10.1016/s0167-4889(97)00138-9

[8] Stinchcombe JC, Page LJ, Griffiths GM. Secretory lysosome biogenesis in cytotoxic T lymphocytes from normal and Chediak Higashi syndrome patients. Traffic. 2000;1: 435–444. 11208129 10.1034/j.1600-0854.2000.010508.x

[9] Olsen I, Bou-Gharios G, Abraham D. The activation of resting lymphocytes is accompanied by the biogenesis of lysosomal organelles. Eur J Immunol. 1990;20: 2161–2170. 10.1002/eji.1830201003 2173661

[10] Sanchez-Ruiz Y, Valitutti S, Dupre L. Stepwise maturation of lytic granules during differentiation and activation of human CD8+ T lymphocytes. PloS One. 2011;6: e27057 10.1371/journal.pone.0027057 22073254 PMC3208563

[11] Griffiths GM, Isaaz S. Granzymes A and B are targeted to the lytic granules of lymphocytes by the mannose-6-phosphate receptor. J Cell Biol. 1993;120: 885–896. 8432729 10.1083/jcb.120.4.885 PMC2200067

[12] Griffiths G, Hoflack B, Simons K, Mellman I, Kornfeld S. The mannose 6-phosphate receptor and the biogenesis of lysosomes. Cell. 1988;52: 329–341. 2964276 10.1016/s0092-8674(88)80026-6

[13] de Wit H, Walter AM, Milosevic I, Gulyas-Kovacs A, Riedel D, Sorensen JB, et al Synaptotagmin-1 docks secretory vesicles to syntaxin-1/SNAP-25 acceptor complexes. Cell. 2009;138: 935–946. 10.1016/j.cell.2009.07.027 19716167

[14] Koch H, Hofmann K, Brose N. Definition of Munc13-homology-domains and characterization of a novel ubiquitously expressed Munc13 isoform. Biochem J. 2000;349: 247–253. 10861235 10.1042/0264-6021:3490247 PMC1221144

[15] Becherer U, Rettig J. Vesicle pools, docking, priming, and release. Cell Tissue Res. 2006;326: 393–407. 10.1007/s00441-006-0243-z 16819626

[16] Ménasché G, Pastural E, Feldmann J, Certain S, Ersoy F, Dupuis S, et al Mutations in RAB27A cause Griscelli syndrome associated with haemophagocytic syndrome. Nat Genet. 2000;25: 173–176. 10.1038/76024 10835631

[17] Feldmann J, Callebaut I, Raposo G, Certain S, Bacq D, Dumont C, et al Munc13-4 is essential for cytolytic granules fusion and is mutated in a form of familial hemophagocytic lymphohistiocytosis (FHL3). Cell. 2003;115: 461–473. 14622600 10.1016/s0092-8674(03)00855-9

[18] Pasche M, Matti U, Hof D, Rettig J, Becherer U. Docking of LDCVs Is Modulated by Lower Intracellular [Ca2+] than Priming. Spafford JD, editor. PLoS ONE. 2012;7: e36416 10.1371/journal.pone.0036416 22590540 PMC3349663

[19] Nofal S, Becherer U, Hof D, Matti U, Rettig J. Primed vesicles can be distinguished from docked vesicles by analyzing their mobility. J Neurosci. 2007;27: 1386–1395. 10.1523/JNEUROSCI.4714-06.2007 17287513 PMC6673599

[20] Dudenhöffer-Pfeifer M, Schirra C, Pattu V, Halimani M, Maier-Peuschel M, Marshall MR, et al Different Munc13 isoforms function as priming factors in lytic granule release from murine cytotoxic T lymphocytes. Traffic Cph Den. 2013;14: 798–809. 10.1111/tra.12074 23590328

[21] Pattu V, Qu B, Marshall M, Becherer U, Junker C, Matti U, et al Syntaxin7 is required for lytic granule release from cytotoxic T lymphocytes. Traffic. 2011;12: 890–901. 10.1111/j.1600-0854.2011.01193.x 21438968

[22] Almers W. Gating currents and charge movements in excitable membranes. Rev Physiol Biochem Pharmacol. 1978;82: 96–190. 356157 10.1007/BFb0030498

[23] Hallermann S, Pawlu C, Jonas P, Heckmann M. A large pool of releasable vesicles in a cortical glutamatergic synapse. Proc Natl Acad Sci. 2003;100: 8975–8980. 10.1073/pnas.1432836100 12815098 PMC166423

[24] Moser T, Neher E. Estimation of mean exocytic vesicle capacitance in mouse adrenal chromaffin cells. Proc Natl Acad Sci U S A. 1997;94: 6735–6740. 9192634 10.1073/pnas.94.13.6735 PMC21227

[25] Halimani M, Pattu V, Marshall MR, Chang HF, Matti U, Jung M, et al Syntaxin11 serves as a t-SNARE for the fusion of lytic granules in human cytotoxic T lymphocytes. Eur J Immunol. 2014;44: 573–584. 10.1002/eji.201344011 24227526

[26] Hartmann J, Scepek S, Lindau M. Regulation of granule size in human and horse eosinophils by number of fusion events among unit granules. J Physiol. 1995;483 (Pt 1): 201–209. 7776232 10.1113/jphysiol.1995.sp020578 PMC1157882

[27] Neher E, Marty A. Discrete changes of cell membrane capacitance observed under conditions of enhanced secretion in bovine adrenal chromaffin cells. Proc Natl Acad Sci U S A. 1982;79: 6712–6716. 6959149 10.1073/pnas.79.21.6712 PMC347199

[28] Schwarz EC, Kummerow C, Wenning AS, Wagner K, Sappok A, Waggershauser K, et al Calcium dependence of T cell proliferation following focal stimulation. Eur J Immunol. 2007;37: 2723–2733. 10.1002/eji.200737039 17899547

[29] Shaner NC, Steinbach PA, Tsien RY. A guide to choosing fluorescent proteins. Nat Methods. 2005;2: 905–909. 10.1038/nmeth819 16299475

[30] Choudhuri K, Dustin ML. Signaling microdomains in T cells. FEBS Lett. 2010;584: 4823–4831. 10.1016/j.febslet.2010.10.015 20965175 PMC3870024

[31] Gillis KD. Techniques for Membrane Capacitance Measurements. Single-Channel Recording. 2nd ed. Plenum; 1995. pp. 155–198.

[32] Henkel AW, Almers W. Fast steps in exocytosis and endocytosis studied by capacitance measurements in endocrine cells. Curr Opin Neurobiol. 1996;6: 350–357. 10.1016/S0959-4388(96)80119-X 8794084

[33] Martina JA, Wu XS, Catalfamo M, Sakamoto T, Yi C, Hammer JA. Imaging of lytic granule exocytosis in CD8+ cytotoxic T lymphocytes reveals a modified form of full fusion. Cell Immunol. 2011;271: 267–279. 10.1016/j.cellimm.2011.07.004 21843881 PMC3407469

[34] Bertrand F, Muller S, Roh K-H, Laurent C, Dupre L, Valitutti S. An initial and rapid step of lytic granule secretion precedes microtubule organizing center polarization at the cytotoxic T lymphocyte/target cell synapse. Proc Natl Acad Sci. 2013;110: 6073–6078. 10.1073/pnas.1218640110 23536289 PMC3625254

[35] Grossman WJ, Verbsky JW, Tollefsen BL, Kemper C, Atkinson JP, Ley TJ. Differential expression of granzymes A and B in human cytotoxic lymphocyte subsets and T regulatory cells. Blood. 2004;104: 2840–2848. 10.1182/blood-2004-03-0859 15238416

[36] Hogan PG, Lewis RS, Rao A. Molecular basis of calcium signaling in lymphocytes: STIM and ORAI. Annu Rev Immunol. 2010;28: 491–533. 10.1146/annurev.immunol.021908.132550 20307213 PMC2861828

[37] Pores-Fernando AT, Zweifach A. Calcium influx and signaling in cytotoxic T-lymphocyte lytic granule exocytosis. Immunol Rev. 2009;231: 160–173. 10.1111/j.1600-065X.2009.00809.x 19754896

[38] Dustin ML, Long EO. Cytotoxic immunological synapses. Immunol Rev. 2010;235: 24–34. 10.1111/j.0105-2896.2010.00904.x 20536553 PMC2950621

[39] Pores-Fernando AT, Bauer RA, Wurth GA, Zweifach A. Exocytic responses of single leukaemic human cytotoxic T lymphocytes stimulated by agents that bypass the T cell receptor. J Physiol. 2005;567: 891–903. 10.1113/jphysiol.2005.089565 16020463 PMC1474224

[40] Stinchcombe JC, Bossi G, Booth S, Griffiths GM. The immunological synapse of CTL contains a secretory domain and membrane bridges. Immunity. 2001;15: 751–761. 11728337 10.1016/s1074-7613(01)00234-5

[41] Marshall MR, Pattu V, Halimani M, Maier-Peuschel M, Muller M-L, Becherer U, et al VAMP8-dependent fusion of recycling endosomes with the plasma membrane facilitates T lymphocyte cytotoxicity. J Cell Biol. 2015;210: 135–151. 10.1083/jcb.201411093 26124288 PMC4493996

[42] Matti U, Pattu V, Halimani M, Schirra C, Krause E, Liu Y, et al Synaptobrevin2 is the v-SNARE required for cytotoxic T-lymphocyte lytic granule fusion. Nat Commun. 2013;4: 1439 10.1038/ncomms2467 23385584

[43] Bou-Gharios G, Moss J, Olsen I. Localization of lysosomal antigens in activated T-lymphocytes. Histochem J. 1991;23: 474–482. 1743996 10.1007/BF01041378

[44] Jahn R, Scheller RH. SNAREs—engines for membrane fusion. Nat Rev Cell Biol. 2006;7: 631–643. 10.1038/nrm2002 16912714

[45] Oheim M, Stühmer W. Tracking chromaffin granules on their way through the actin cortex. Eur Biophys J EBJ. 2000;29: 67–89. 10877017 10.1007/s002490050253

[46] Oheim M, Loerke D, Stühmer W, Chow RH. The last few milliseconds in the life of a secretory granule. Docking, dynamics and fusion visualized by total internal reflection fluorescence microscopy (TIRFM). Eur Biophys J EBJ. 1998;27: 83–98. 9530824 10.1007/s002490050114

[47] de Wit H, Cornelisse LN, Toonen RF, Verhage M. Docking of secretory vesicles is syntaxin dependent. PloS One. 2006;1: e126 10.1371/journal.pone.0000126 17205130 PMC1762430

[48] Toonen RF, Kochubey O, de Wit H, Gulyas-Kovacs A, Konijnenburg B, Sorensen JB, et al Dissecting docking and tethering of secretory vesicles at the target membrane. EMBO J. 2006;25: 3725–3737. 10.1038/sj.emboj.7601256 16902411 PMC1553188

[49] Brown ACN, Oddos S, Dobbie IM, Alakoskela J-M, Parton RM, Eissmann P, et al Remodelling of cortical actin where lytic granules dock at natural killer cell immune synapses revealed by super-resolution microscopy. PLoS Biol. 2011;9: e1001152 10.1371/journal.pbio.1001152 21931537 PMC3172219

[50] Krzewski K, Coligan JE. Human NK cell lytic granules and regulation of their exocytosis. Front Immunol. 2012;3: 335 10.3389/fimmu.2012.00335 23162553 PMC3494098

[51] Billadeau DD. T cell activation at the immunological synapse: vesicles emerge for LATer signaling. Sci Signal. 2010;3: pe16 10.1126/scisignal.3121pe16 20460646

[52] Blott EJ, Griffiths GM. Secretory lysosomes. Nat Rev Mol Cell Biol. 2002;3: 122–131. 10.1038/nrm732 11836514

[53] Colvin RA, Means TK, Diefenbach TJ, Moita LF, Friday RP, Sever S, et al Synaptotagmin-mediated vesicle fusion regulates cell migration. Nat Immunol. 2010;11: 495–502. 10.1038/ni.1878 20473299 PMC2951881

[54] Soares H, Henriques R, Sachse M, Ventimiglia L, Alonso MA, Zimmer C, et al Regulated vesicle fusion generates signaling nanoterritories that control T cell activation at the immunological synapse. J Exp Med. 2013;210: 2415–2433. 10.1084/jem.20130150 24101378 PMC3804939

[55] Qu B, Pattu V, Junker C, Schwarz EC, Bhat SS, Kummerow C, et al Docking of Lytic Granules at the Immunological Synapse in Human CTL Requires Vti1b-Dependent Pairing with CD3 Endosomes. J Immunol. 2011;186: 6894–6904. 10.4049/jimmunol.1003471 21562157

[56] Peters PJ, Geuze HJ, Van der Donk HA, Slot JW, Griffith JM, Stam NJ, et al Molecules relevant for T cell-target cell interaction are present in cytolytic granules of human T lymphocytes. Eur J Immunol. 1989;19: 1469–1475. 10.1002/eji.1830190819 2789142

[57] Becherer U, Medart MR, Schirra C, Krause E, Stevens D, Rettig J. Regulated exocytosis in chromaffin cells and cytotoxic T lymphocytes: How similar are they? Cell Calcium. 2012;52: 303–312. 10.1016/j.ceca.2012.04.002 22560267

