# Supplementary figures and images for: Behavior and Properties of Mature Lytic Granules at the Immunological Synapse of Human Cytotoxic T Lymphocytes
Source: PLoS One. 2015 Aug 21;10(8):e0135994. doi: 10.1371/journal.pone.0135994 (PMC4546685; doi:10.1371/journal.pone.0135994)

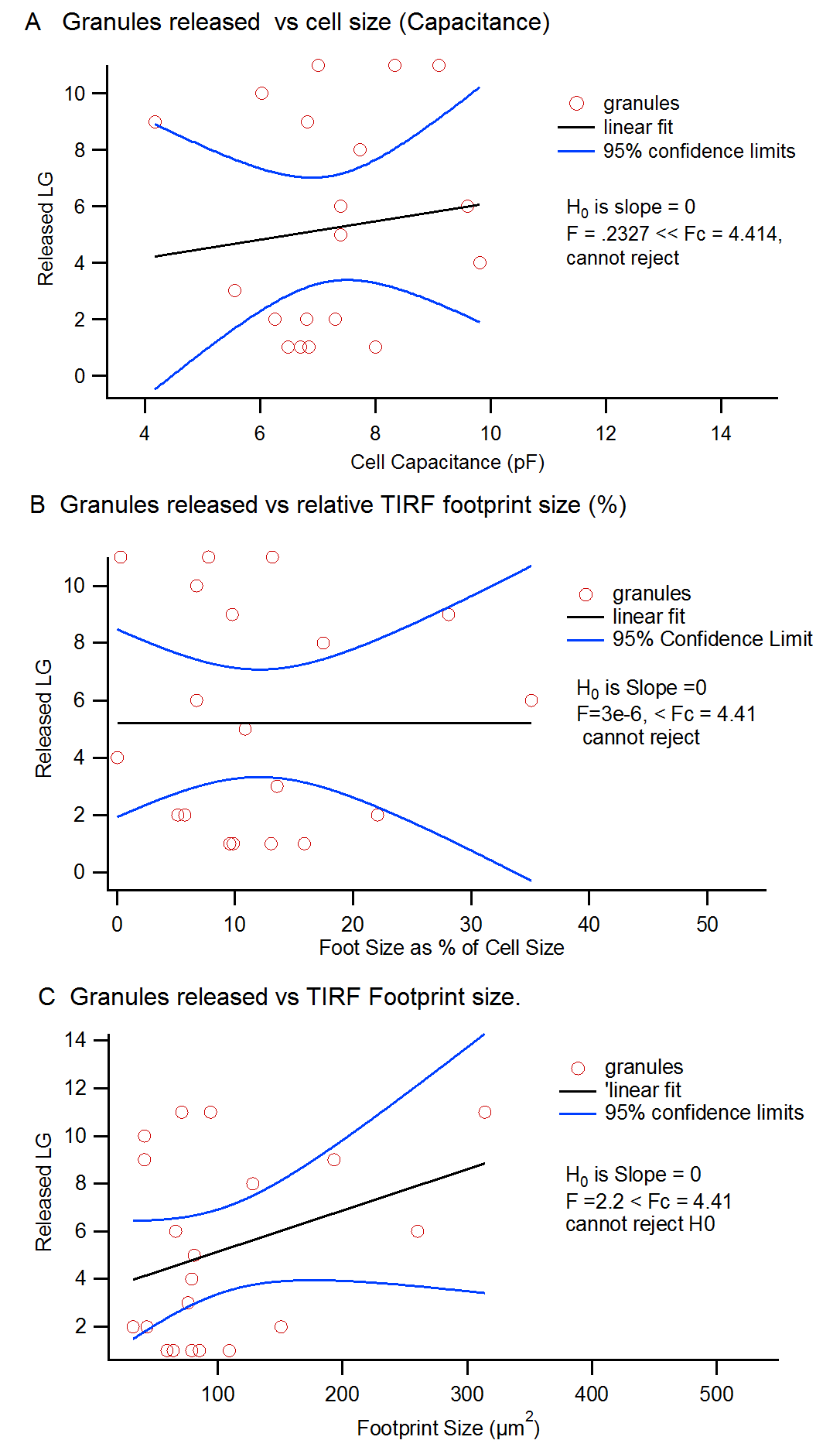

Supplement: S1 Fig — The number of released granules is plotted vs these three variables. (TIF) [file pone.0135994.s001.tif]
